# Which patients to sample in clinical cohort studies when the number of events is high and measurement of additional markers is constrained by limited resources

**DOI:** 10.1002/cam4.3381

**Published:** 2020-08-19

**Authors:** Dominic Edelmann, Kristin Ohneberg, Natalia Becker, Axel Benner, Martin Schumacher

**Affiliations:** ^1^ Division of Biostatistics German Cancer Research Center Heidelberg Germany; ^2^ Institute for Medical Biometry and Statistics Faculty of Medicine and Medical Center University of Freiburg Freiburg Germany; ^3^ Max Rubner‐Institute Federal Research Institute of Nutrition and Food Karlsruhe Germany

**Keywords:** case‐cohort, nested case‐control, survival

## Abstract

**Purpose:**

We consider an existing clinical cohort with events but limited resources for the investigation of a further potentially expensive marker. Biological material of the patients is stored in a biobank, but only a limited number of samples can be analyzed with respect to the marker. The question arises as to which patients to sample, if the number of events preclude standard sampling designs.

**Methods:**

Modifications of the nested case‐control and the case‐cohort design for the proportional hazards model are applied, that allow efficient sampling in situations where standard nested case‐control and case‐cohort are not feasible. These sampling designs are compared to simple random sampling and extreme group sampling, the latter including only patients with extreme outcomes, ie either with an event early in time or without an event until at least a point later in time.

**Results:**

The modified nested case‐control design and the modified case‐cohort design provide powerful methods for sampling in a clinical cohort with many events. The simple random sampling usually is less efficient. If focus is on precise estimation of a potential effect in terms of a hazard ratio, extreme group sampling is not competitive. If focus is on screening for important biomarkers, extreme group sampling markedly outperforms the other sampling designs.

**Conclusions:**

When it is not feasible to sample all events, a modified nested case‐control design or case‐cohort design leads to efficient effect estimates in the proportional hazards model. If screening for important biomarkers is the primary objective, extreme group sampling is preferable.

## Graphical abstract

The article focuses on which patients to sample in clinical cohort studies when the number of events is high and measurement of additional markers is constrained by limited resources. In such a setting modifications of the nested case‐control design or the case‐cohort design provide powerful methods. The simple random sampling usually is less efficient and the so called extreme group sampling (comparing patients with an early event with long‐term survivors) is preferable if screening for important biomarkers is the primary objective.

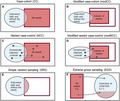

## 1 INTRODUCTION

In clinical cancer research, we are often faced with the following situation: baseline and follow‐up data (such as overall survival or disease‐free survival) of a clinical cohort are available and tissue or blood of the patients is stored in a biobank. Yet the measurement of additional markers of interest is constrained by limited resources. As a consequence, only a subset of patients can be included when analyzing the association of these markers and the available time‐to‐event data.

In epidemiologic research, standard approaches in such a situation are sampling designs, such as nested case‐control (NCC) or case‐cohort (CC) designs. These outcome‐dependent sampling designs include all the patients with an event, but only a subset of the patients without the event. These sampling designs have been motivated from situations when the event of interest is rare.^1^, ^2^

Our situation is fundamentally different in that the event of interest is usually common, such as deaths, recurrences, or progressions of the disease. Due to cost restrictions, it is then often not possible to include all events making standard NCC and CC infeasible. Consequently, modifications of these sampling designs are needed, that are partially available in the statistical literature(p.75),^3,4^ but rarely used in practice.

In addition, ad‐hoc approaches for sampling in the situation of nonrare events are proposed such as simple random sampling and extreme group sampling.^5^, ^6^ Simple random sampling just draws a random sample of the entire cohort without considering the event status of the individuals. The rationale behind the extreme group sampling is that individuals with extreme outcomes are compared: individuals that experience the event within an early time interval are compared to those individuals that do not experience the event before a given time point later in time.

Our aim is to evaluate different sampling designs in the case of a (clinical) cohort where the total number of events excludes standard approaches such as an NCC or CC design. We consider modifications of NCC and CC, as well as simple random sampling and extreme group sampling. These cohort sampling designs are evaluated both in terms of their potential for precise estimation (eg. a hazard ratio) as well as their ability to detect relevant biomarkers in a screening procedure.

The manuscript is structured as follows: In Section 2 ‘Materials and Methods’, the clinical cohort data examples (2.1), the different cohort sampling designs under consideration (2.2) as well as the methods of the statistical analysis are presented. In Section 3 we provide the results of analyzing the original full cohort (3.1) as well as those after applying the different sampling designs (3.2). Additionally, we provide results on the power of the designs when the focus is on testing (3.3) and on the inflation of the standard error that is coming along with a strongly decreased sample size (3.4). We finish the manuscript with a discussion of the obtained results in Section 4.

## 2 MATERIALS AND METHODS

### 2.1 Data examples

#### 2.1.1 DACHS study

In this paper, we use the data of the DACHS study (DACHS: Darmkrebs: Chancen der Verhütung durch Screening/Colorectal cancer: chances for prevention through screening) to illustrate the application of various sampling designs. The patient cohort consists of the cases from a population‐based case‐control study on colorectal cancer (CRC) in Germany where cases had undergone an additional long‐term follow‐up (median follow‐up 4.97 years) with regard to relapse‐free and overall survival. In this article, we focus on the relapse‐free survival of cases. Further details on the study can be found elsewhere.^7^, ^8^, ^9^ Basic patient and tumor characteristics such as age, gender, UICC stage, and adjuvant chemotherapy (yes/no) are available for all patients; in addition, various specific factors have been measured for a large proportion of patients in routinely stored tumor tissue. One of these factors is the so‐called Microsatellite instability (MSI) that is defined as hypermutability of microsatellite sequences. MSI occurs in many human cancers and results from an inactivation of the DNA mismatch repair system.^10^ This factor is classified as MSI‐high or MSI‐low and MSS (microsatellite stable) combined (in the following “MSI low/no”).^11^

MSI status is available in N = 1550 patients, and since this factor will play the role of an “expensive” covariate in our illustration, these 1550 patients form the “full cohort” in the sequel. We report the basic characteristics of the patients in this cohort in Table 1. The outcome of interest is relapse‐free survival (RFS), which is defined as the length of time after primary treatment for a cancer until new signs or symptoms of that cancer or death, whatever occurs first. Relapse or death is observed for a total of 569 patients (569/1550 = 36.7%). We fit a Cox proportional hazard regression for the association of the MSI status with RFS and adjust for age, gender, UICC stage, and administration of chemotherapy.

**TABLE 1:** Results of a Cox analysis of all covariates considered in the original DACHS study (upper table A) and in the original GBSG data (lower table B)

DACHS cohort (N = 1550)			log (HR)	SE	HR	95% CI
(A)						
Age, 1‐year increase	Mean (range)	68.6 (33.0‐94.0)	0.019	0.004	1.02	[1.01,1.03]
Sex	Female	889 (57.4%)	0	—	1	—
	Male	661 (42.6%)	−0.002	0.086	1.00	[0.84,1.18]
UICC Cancer Stage	1	289 (18.6%)	0	—	1	—
	2 vs 1	511 (33.0%)	0.490	0.168	1.63	[1.17,2.27]
	3 vs 1	533 (34.4%)	1.150	0.182	3.16	[2.21,4.51]
	4 vs 1	217 (14.0%)	2.776	0.197	16.05	[10.91,23.62]
Adjuvant chemotherapy	No	830 (53.5%)	0	—	1	—
	Yes	720 (46.5%)	−0.224	0.126	0.80	[0.63,1.02]
Microsatellite instability	Low/no	1404 (90.6%)	0	—	1	—
	High	146 (9.4%)	−0.229	0.179	0.80	[0.56,1.13]

GBSG cohort (N = 686)			log (HR)	SE	HR	95% CI
(B)						
Age, 1‐year increase	Mean (range)	53 (21.0‐80.0)	−0.005	0.009	0.99	[0.98,1.01]
NPI	<5.4	385 (56.1%)	0	—	1	—
	>5.4	301 (43.9%)	0.652	0.121	1.92	[1.51,2.43]
Menopausal status	No	290 (42.3%)	0	—	1	—
	Yes	396 (57.7%)	0.187	0.184	1.21	[0.84,1.73]
Oestrogen receptor	Mean (range)	96 (0.0‐1144.0)	0.0002	0.0005	1.00	[0.999,1.001]
Tamoxifen	No	440 (64.1%)	0	—	1	—
	Yes	246 (35.9%)	−0.374	0.129	0.69	[0.53,0.89]
Progesterone receptor	<20 fmol	269 (39.2%)	0	—	1	—
	>20 fmol	417 (60.8%)	−0.630	0.129	0.53	[0.41,0.69]

95% CI, 95% confidence interval for the HR; HR, hazard ratio; log(HR), log hazard ratio; SE, standard error of the log(HR).

#### 2.1.2 German breast cancer study

Our second example is a data set provided by the German Breast Cancer Study Group (GBSG).^12^ The data set contains 686 patients with primary node positive breast cancer. The event of interest is relapse‐free survival (RFS). Relapse or death is observed for 299 patients. The median follow‐up in the GBSG dataset is 4.5 years. For illustration we consider the progesterone receptor status (pgr) as the ‘expensive’ covariate. We fit a Cox proportional hazards model for the association of the pgr (pgr ≥ 20 fmol vs pgr < 20 fmol) with RFS and adjust for hormonal treatment with tamoxifen, age, estrogen receptor status, menopausal status as well as for the Nottingham Prognostic Index (NPI) that is defined as.

$$\text{NPI}=0.02\times \text{size}\left(\text{in mm}\right)+\text{lymph node stage}+\text{tumor grade}$$

where lymph node stage is equal to 1 for node‐negative patients, 2 for patients with one to three positive lymph nodes, and 3 if four or more lymph nodes were involved.

### 2.2 Sampling designs

#### 2.2.1 Nested case‐control and case‐cohort design

In the sequel, we pretend that there are not sufficient resources available to measure MSI status in the DACHS cohort and pgr in the GBSG data for all patients, but only for a certain portion (we have therefore called MSI and pgr status an “expensive covariates”). Then the question arises which sample from the cohort of patients to take and how this can be done in an efficient way. In epidemiology, the nested case‐control study (NCC) design and the case‐cohort study (CC) have been proposed.^1^, ^2^ Briefly, in the NCC design all patients with events are included (the “cases”) and for each “case”, one or more “controls” are sampled from the corresponding risk sets, ie all patients that have not yet experienced an event at the event time of the “case” (so‐called incidence density sampling^13^).

**FIGURE 1:**
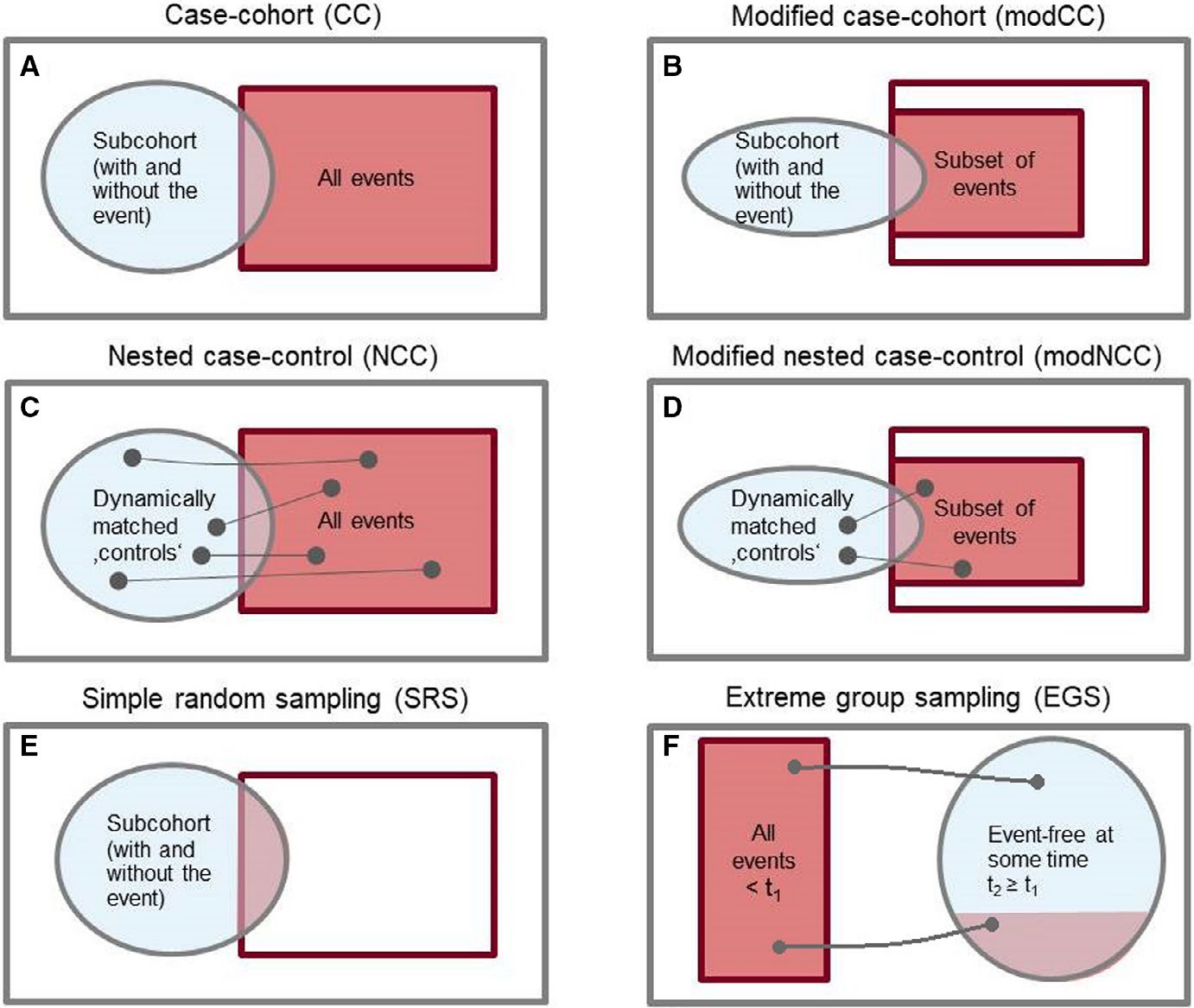
Schematic illustration of sampling designs

The CC design consists of two steps: first, a randomly chosen subset of the cohort (the “subcohort”, usually of the same size as the set of “cases”) is sampled which consists of patients with, as well as without, an event. The subcohort is extended by the remaining cases in the second step. These two designs are schematically displayed in Figure 1A and C and can lead to considerable savings of resources when the event of interest is rare.^1^, ^2^

#### 2.2.2 Modified nested case‐control and modified case‐cohort design

In the DACHS study, however, the number of events is 569 (for a total of 1550 individuals); so with a CC design one samples at least all the cases (plus a subcohort of a desired size) and a NCC design would lead to about twice of the number of events that is not much less than the full cohort. Thus, modifications of the two sampling designs are required to cope with the situation of a common event that is typical when clinical cohorts are investigated. Such modifications are available in the statistical literature(p.75)^3,4^ and we describe two of those. The NCC design can be modified (therefore called modNCC) in that only a certain portion of “cases” is randomly chosen with a pre‐specified sampling probability, say *P*. For each sampled “case” (patient with event) a control patient is sampled from the corresponding risk set as in the ordinary NCC sampling design. The modification of the CC sampling design (modCC) is also straightforward: a (reduced) subcohort is randomly chosen and only a subset of the remaining cases is sampled, leading to a reduced sample size. These two modifications are schematically illustrated in Figure 1B and D.

#### 2.2.3 Simple random sampling and extreme group sampling

We will also consider two other sampling designs that are sometimes used.^5^, ^6^ The first is simple random sampling (SRS) where just a certain fraction of patients is sampled from the full cohort irrespective of their event status. The second is called extreme group sampling (EGS) where all patients with an early event (smaller than some time *t*_1_) and a subset of all patients that are event free at some time *t*_2_ ≥ *t*_1_ are sampled; all patients that are censored before *t*_2_ or observe an event in [*t*_1,_ *t*_2_) are excluded. For each “case” (event time smaller than *t*_1_), we will draw one “control” (event‐free at *t*_2_).

We implement two different versions of EGS in our data examples; for the first we set *t*_2_ = *t*_1_ and for the second *t*_2_ = 5 years was chosen. In real applications *t*_1_ (just like *t*_2_) would be typically chosen as a clinically meaningful boundary. In our data examples the median follow‐up is about 5 years in both cohorts. In this article, to enable a fair comparison, *t*_1_ needs to be varied for the single implementations of EGS to obtain the same sample sizes as for the other sampling designs. Depending on the available resources and version, the used values for *t*_1_ vary from 10 to 15 months for the DACHS data and from 14 to 18 months for the GBSG data.

SRS and EGS are illustrated in Figure 1E and F. Although SRS yields unbiased results, it is not efficient^2^ and should only be used in exceptional situations, eg when the conduct of other sampling designs is not feasible. We include SRS for comparison reasons and because we found that SRS is applied in practice.^5^

### 2.3 Statistical analysis

#### 2.3.1 Analysis methods

We fit an ordinary Cox proportional hazard regression for the event of interest (relapse‐free survival for the DACHS cohort as well as for the GBSG data) including the ‘expensive’ covariate (MSI status for the DACHS cohort and pgr for the GBSG data) and adjust for baseline covariates as described in Section 2.1.

For the NCC and modNCC data instead of using a stratified Cox regression (the strata being the matched sets) resembling a conditional logistic regression we reuse the sampled controls at all observed event times when they are at risk and hence use a weighted Cox regression including Kaplan‐Meier based weights.^14^ For the CC and modCC approach a weighted Cox regression including so‐called “inverse sampling probability weighting”^15^ is applied, that is readily implemented for the analysis in a Cox regression model.

The data obtained by SRS is analyzed by a standard Cox regression. Straightforward application (that means without any weighting or further consideration of the sampling in the analysis step) of EGS might lead to biased results in terms of the hazard ratio as compared to a full cohort analysis. An involved likelihood‐based (“conditional”) approach is presented^16^, ^17^ to reduce this bias. We used this approach in our later data analysis.

For testing the association of biomarkers with survival, a Wald test is applied for the full cohort and all sampling designs except for EGS using a robust estimate^18^ of the variance of the regression coefficient. For testing the association of biomarkers with survival, a Wald test based on a simple logistic regression is used for the EGS design treating individuals with event time smaller than *t*_1_ as cases and individuals that are still alive at time *t*_2_ as controls.^17^ The variance estimate for this logistic regression is obtained by maximum likelihood.

#### 2.3.2 Comparison of bias and variance of estimated hazard ratios

In order to compare the bias and variance of the estimated hazard ratios obtained by different sampling designs, we repeatedly performed these sampling processes (modNCC, modCC, EGS, SRS) using a resampling approach that will be outlined below. To obtain a fair comparison the parameters of the sampling designs were chosen to achieve approximately the same number of sampled patients N. Three different sample sizes were considered. For the DACHS cohort these are.

- high resource setting N = 924 (corresponding to a standard NCC design with 1:1 matching).
- medium resource setting N = 418 (modNCC 1:1, case sampling probability *P* = .4).
- low resource setting N = 270 (modNCC 1:1, *P* = .25).

For quantifying the bias of the different estimators and the variation, which is due to the sampling procedure, we repeatedly (*M* = 10 000 times) applied each sampling design on the DACHS cohort and GBSG cohort respectively. For each repetition and sampling design, we fit a Cox proportional hazard regression for relapse‐free survival including the MSI status (always adjusted for age, gender, stage, and administration of chemotherapy) for the DACHS cohort and pgr (always adjusting for hormonal treatment with tamoxifen, age, estrogen receptor status, menopausal, and NPI) for the GBSG data and present the mean regression coefficient (column “log(HR)”) and mean hazard ratio (column “HR”). For quantifying the bias of the estimators, the full cohort estimate is considered as gold standard. For quantifying the standard error due to sampling, we evaluated the standard error of the coefficient estimates over all *M* runs, which is provided in the column “sampSE”.

For a reliable estimate of the total variation (variation in full cohort + variation due to sampling), we pursued a bootstrap^19^ approach. This will create 10,000“bootstrapped cohorts” of the same size as the original cohort by sampling with replacement from the original full cohort. Then each design was applied once on each of the 10 000 “bootstrapped cohorts”. The total empirical standard error is then evaluated as the standard error of the estimates based on the sampling designs (using the same models as described above) over all 10 000 ‘bootstrap cohorts’ and is given in the column “totalSE”. The empirical error for the full cohort was obtained as the standard error of the estimates over all 10 000 ‘bootstrapped cohorts’ (without applying any sampling design).

#### 2.3.3 Inflation factor of the modNCC design

As we focus on parameter estimates with standard errors, we consider the loss in precision in terms of an inflation factor (IF) of the standard error, that is defined as

$$\text{IF}=\frac{\text{totalSE}(\text{sampling}\;\text{design})}{\text{totalSE}(\text{full}\;\text{cohort})}$$

with the total empirical standard error using the sampling design in the nominator and the total empirical standard error using the full cohort in the denominator.

For the modNCC design it is possible to obtain an analytic expression as an approximation of this inflation factor, which is given by $1/\sqrt{pm/\left(m+1\right)}$, where *m* is the number of controls matched to each case and *p* the fraction of sampled cases (see also^2^). As for the modNCC design usually *m* = 1, the inflation factor simplifies to $1/\sqrt{p/2}$. Using a case sampling probability of *p* = 0.5 hence yields an inflation factor of $1/\sqrt{0.5/2}=2$. Using this simple formula it is possible to assess the loss in precision coming along with the reduced data set prior to analysis. To be precise, the formula for the inflation factor actually focuses on a modNCC design analyzed by a stratified regression (the strata being the matched sets). Re‐using the controls at all times when they are at risk and using a weighted regression (as described in Section ‘2.3 Statistical analysis’) yields a reduced standard error as compared to a stratified analysis. Hence, the formula presented above can be considered as an upper bound for the inflation of the standard error using a modNCC design as compared to the full cohort.

#### 2.3.4 Comparison of the power to detect an important covariate

While MSI is not significant for relapse‐free survival, pgr is highly significant in the GBSG dataset (see Table 1B). We can thus (quite) safely conclude that pgr is indeed associated with relapse‐free survival.

For the purpose of assessing the performance of a specific sampling design for detecting an important covariate, we tested for the association of pgr with relapse‐free survival in all *M* = 10 000 subsamples obtained by repeatedly applying the same sampling design on the original cohort (see first paragraph of Section 2.3.2). The “power” of a sampling design is then obtained by the fraction of subsamples in which pgr was significant.

We note that, technically, this is not a power comparison in the proper meaning of the word (since one can never be entirely sure from real data that there is an indeed a true association). However, as already mentioned above, considering the high significance of pgr in the full cohort, the performed analysis is very similar to a proper power comparison.

## 3 RESULTS

### 3.1 Original cohort

The results of a standard analysis with the Cox regression model with respect to relapse‐free survival for the DACHS cohort are displayed in Table 1A (upper table). We see that age and stage have a significant effect on relapse‐free survival, gender has no effect at all, and administration of chemotherapy has a borderline independent effect (which is not surprising since it is related to stage). Our primary interest, however, is estimating the hazard ratio of MSI. Here, patients with high MSI have a slightly reduced risk of relapse or death compared to those with no MSI or low levels as indicated by a hazard ratio of 0.80. The 95%‐confidence interval ranges from 0.56 to 1.13. As described above we consider the full cohort estimates as the gold standard when evaluating different sampling designs. The results mentioned in the paragraph above are in accordance with results from other recent studies^20^, ^21^ and also with a related analysis of the DACHS study.^10^

The results of the original GBSG cohort analyzed by a Cox regression are given in Table 1B (lower table). Patients with progesterone receptor ≥ 20 fmol have a reduced risk of relapse or death (hazard ratio 0.53 with 95% confidence interval [0.41,0.69]) as compared to patients with progesterone receptor < 20 fmol.

### 3.2 Application of sampling designs

The results when repeatedly applying the sampling designs to the full cohort are summarized in Table 2, showing the average log hazard ratio for the covariate under consideration together with the corresponding standard error due to sampling (“sampSE”) and the total standard error (“totalSE”).

**TABLE 2:** Mean results of different sampling for the DACHS cohort (adjusted HR for MSI, upper table A) and the GBSG cohort (adjusted HR for pgr, lower table B)

DACHS	n.sample	n.event	log(HR)	HR	sampSE	totalSE	IF
(A)							
Full cohort	1550	569	−0.229	0.80		0.180	1.00
NCC	924	569	−0.220	0.80	0.139	0.229	1.27
CC	925	569	−0.219	0.80	0.142	0.231	1.28
SRS	924	339	−0.237	0.79	0.152	0.238	1.32
modNCC	418	255	−0.225	0.80	0.291	0.343	1.91
modCC	418	236	−0.230	0.79	0.290	0.350	1.94
SRS	418	154	−0.245	0.78	0.360	0.407	2.26
EGS *t*_2_ = *t*_1_	419	276	−0.251	0.78	0.372	0.520	2.89
EGS *t*_2_ = 5y	419	247	−0.542	0.58	0.360	0.506	2.81
modNCC	270	165	−0.228	0.80	0.398	0.441	2.45
modCC	270	157	−0.223	0.80	0.401	0.446	2.48
SRS	270	99	−0.267	0.77	0.434	0.477	2.65
EGS *t*_2_ = *t*_1_	271	181	−0.542	0.58	0.589	0.827	4.59
EGS *t*_2_ = 5y	273	153	−0.504	0.60	0.482	0.726	4.03

GBSG	n.sample	n.event	log(HR)	HR	sampSE	totalSE	IF	Power
(B)								
Full cohort	686	299	−0.630	0.53		0.134	1.00	
NCC	454	299	−0.635	0.53	0.099	0.165	1.23	0.999
CC	455	299	−0.637	0.53	0.113	0.177	1.31	0.996
SRS	454	198	−0.630	0.53	0.096	0.164	1.22	0.996
modNCC	213	139	−0.624	0.54	0.208	0.252	1.89	0.767
modCC	213	126	−0.632	0.53	0.214	0.256	1.91	0.760
SRS	213	93	−0.623	0.54	0.245	0.281	2.10	0.764
EGS *t*_2_ = *t*_1_	213	147	−1.058	0.35	0.285	0.376	2.81	1,000
EGS *t*_2_ = 5y	214	142	−0.830	0.44	0.217	0.322	2.40	1,000
modNCC	139	90	−0.621	0.54	0.281	0.322	2.40	0.545
modCC	139	85	−0.639	0.53	0.298	0.329	2.46	0.557
SRS	139	61	−0.627	0.53	0.286	0.322	2.40	0.553
EGS *t*_2_ = *t*_1_	139	94	−0.370	0.69	0.372	0.462	3.45	0.924
EGS *t*_2_ = 5y	139	88	−0.659	0.52	0.371	0.488	3.64	0.990

no. sample, number of distinct individuals included; no. event, number of events included; log(HR), adjusted log hazard ratio of MSI (DACHS) and pgr (GBSG); HR, adjusted hazard ratio of MSI (DACHS) and pgr (GBSG); sampSE, empirical standard error of the logHR due to sampling design; totalSE, total empirical standard error of the logHR; IF, inflation factor of the standard error defined as IF = totalSE(sampling design)/totalSE(cohort); power, fraction of repetitions, in which the corresponding test (α = 0.05) rejects the null hypothesis of no association; for EGS a simple logistic regression is used.

The results for MSI in the DACHS cohort are provided in Table 2A, the corresponding results for pgr in the GBSG dataset are given in Table 2B. The values for the full cohort (Table 2, average results when repeatedly applying the sampling designs to the full cohort) coincide with those obtained in the analysis of the original data of the full cohort (Table 1) as they should. For the DACHS cohort, the high resource setting (924 individuals included, hence 924/1550 = 60% of the full cohort) corresponds to the usual application of a nested case‐control (NCC) with 1:1 matching (hence one control matched to each case, no case sampling) and a case‐cohort (CC) design where all 569 “cases”, ie patients with event, are included together with either dynamically matched patients at risk for the NCC or a randomly chosen subcohort of corresponding size for the CC design. Both designs give comparable results in terms of estimated hazard ratios; their standard errors are less than 1.3 times higher than those derived from the full cohort. With simple random sampling (SRS), only 339 patients with event are sampled on average leading to a somewhat larger standard error. Since it is not possible to obtain a sample size of approximately 924 with an EGS as described in Section 2.2.3, this sampling design is omitted in the high resource setting.

In the medium resource setting (418 individuals, that is 418/1550 = 27% of the full cohort), the picture is similar but standard errors of modNCC and modCC are about 1.9 times larger than those in the full cohort. SRS again gives larger standard errors (2.30 times larger). With both implementations of the EGS, the total standard errors are larger than for all other designs (2.86/2.75 times larger). Moreover, for the EGS design with *t*_2_ = 5 years, there is a substantial deviation of the average estimated HR from the corresponding estimate from the full cohort, while the average estimated HR for the EGS design with *t*_2_ = *t*_1_ is in good agreement with the full cohort estimate.

The low resource setting (270 individuals) demonstrates the limitations of sampling designs in a clinical cohort study: With only 270 patients included (≈ 17% of the full cohort), the standard errors of the estimated log‐hazard ratios are about 2.5 times larger than that derived from the full cohort with SRS again yielding poorer precision than the modNCC and the modCC sampling design. This would only be acceptable when a very large effect of the “expensive” covariate could be anticipated which is not the case for the effect of MSI status in the DACHS study. For EGS, we observe large standard errors (4.56/4.02 times higher than in the full cohort); moreover the average HR estimates of both implementations show a substantial deviation from the full cohort estimates. Finally, we note that while the differences in inflation factor between modNCC/modCC and SRS are rather subtle, applying more complex sampling designs like modNCC/modCC may lead to substantial savings. In the medium resource setting, the relative efficiency between modNCC and SRS is (2.30/1.92)^2 = 1.44, indicating that roughly 44% more samples would be needed to obtain the same standard error as modNCC with an SRS design. For the small resource setting, the corresponding relative efficiency is (2.66/2.46)^2 = 1.17, implying that an SRS design would require approximately 17% more patients.

For the GBSG data the estimated hazard ratios for the high resource setting (454 individuals, hence 66% of the full cohort) correspond to the full cohort estimate. Also for the medium resource setting (213 individuals, 31% of the full cohort) the estimates are close to the full cohort counterpart for the modNCC, the modCC and SRS, yet the standard errors are about two times higher. The two EGS implementations yield decreased HRs of 0.35 and 0.43, respectively, as compared to the gold standard of 0.53 for the full and show a considerably higher standard error than modNCC, modCC, and SRS.

### 3.3 Power in detecting important covariates

The results for the power comparison in detecting a significant effect of pgr on relapse‐free survival in the GBSG data are summarized in the column “Power” in Table 2B.

These results demonstrate that a logistic regression based on EGS markedly outperforms all other sampling design in terms of power of detecting an important covariate. Notably, for the medium resource setting (213 individuals), the test based on EGS rejects the null hypothesis of no association in every single repetition, while the power of the other sampling designs is below 0.8. The performance of the two different EGS implementations in the low resource setting (139 individuals) with a power of 0.989 and 0.884, respectively, is still substantially better than the performance of the other designs in the medium resource setting. These findings indicate that EGS designs enable substantial saving in situations, where detection of important biomarkers is the primary objective.

### 3.4 Inflation factor of the standard errors

As described in subsection ‘2.3.3 Inflation factor of the modNCC design’ it is possible to calculate an analytic expression as an approximation of the inflation factor of the standard error based on the modNCC design compared to a full cohort analysis. The inflation factor is given by $1/\sqrt{p/2}$, where *p* denotes the case sampling probability. Consider Table 2A with the mean results of the sampling designs for the DACHS cohort. For the modNCC design with a sample size of 418 individuals and 255 events included, we use *P* = .45 and obtain an approximated inflation factor of 2.11, while in fact using the empirical standard errors in Table 2A we find that the empirical inflation factor is 1.92. For modNCC with 270 individuals and 159 events included, we have *P* = .29 and the approximated inflation factor is $1/\sqrt{0.29/2}=2.63$, while in our data example, we find that the empirical inflation factor is 2.46. Hence the predicted inflation factor roughly reflects the increase in the standard error found in the results and thus might help to roughly assess the loss in precision coming along with the savings in resources using a modNCC design as compared to the full cohort.

## 4 DISCUSSION AND RECOMMENDATIONS

For a clinical researcher and/or a statistician involved in clinical cancer research the question arises which sampling design to choose when there are not sufficient resources to measure a new marker for all patients in a cohort, where data on basic characteristics and follow‐up are already available.

We investigate the performance of various sampling designs for the proportional hazards model in data from the DACHS study on colorectal cancer and the GBSG data on breast cancer with long‐term follow‐up with regard to relapse‐free survival. When sufficient resources are available, a classical NCC or CC design yields good results in our examples, both for estimating the HR and for the power of detecting a significant effect.

When resources are not sufficient to sample all cases, our results suggest that the sampling design should be chosen based on the primary goals of the analysis. We investigated two different purposes in this article—estimation of the HR and testing for the effect of a biomarker. For estimating the HR, we conclude based on our data examples that the modNCC and modCC design perform equally well. The estimates provided by SRS present themselves with a slightly increased standard error as compared to the modNCC and modCC design. Yet in the context of limited resources to evaluate an expensive biomarker this loss in efficiency might be relevant. In several cases in our data examples, EGS yields HR estimates that deviate from those obtained by a full cohort. Moreover EGS shows a considerably higher variation in estimating the HR than modNCC and modCC.

For testing the effect of a biomarker, a simple logistic regression based on EGS markedly outperforms the other sampling designs under consideration. Hence, if one considers estimation of the effect of a covariate on survival (ie of a HR), modNCC and modCC designs are a good choice. On the other hand, if screening of important biomarkers is the objective, EGS is preferable.

The main limitation of this work is that we only consider two specific data examples. Both of these data examples involve rather heavy censoring and a binary covariate of interest. To obtain a more detailed understanding of the strengths and weaknesses of the different designs, future work may compare these methods using extensive simulations varying the censoring regime, distribution of the covariate of interest, and other parameters in the model. We also note that some biomarker studies involve high‐dimensional data, which has not been a focus of this article and may also be addressed in the future. Finally, our comparison was restricted to rather simple sampling designs. While these designs are easily comprehensible, there may be further performance gains by using more sophisticated designs, see eg.^15^

The savings of resources (a new marker is measured only in a subsample of the entire cohort) gained by the use of a sampling design comes along with a loss in precision in terms of an inflation of standard errors. For the modNCC design yet it is possible to roughly assess the corresponding inflation factor (2.3.3). This can in turn help to spend the available resources in a most efficient way and/or to decide whether such a sampling approach would be a useful enterprise at all.

## CONFLICT OF INTEREST

The authors declare no potential conflicts of interest.

## AUTHOR CONTRIBUTIONS

Dominic Edelmann was involved in methodology, formal analysis and writing—original draft, review and editing. Kristin Ohneberg was involved in methodology, formal analysis and writing—original draft, review and editing. Natalia Becker was involved in methodology, formal analysis and writing—original draft. Axel Benner was involved in data curation, methodology, writing—review and editing. Martin Schumacherwas involved in methodology, writing—review and editing.

## Data Availability

The GBSG data that support the findings of this study are openly available at http://mfp.imbi.uni‐freiburg.de/book, Table A.2 "GBSG breast cancer”. Data of the DACHS cohort is not openly available.
